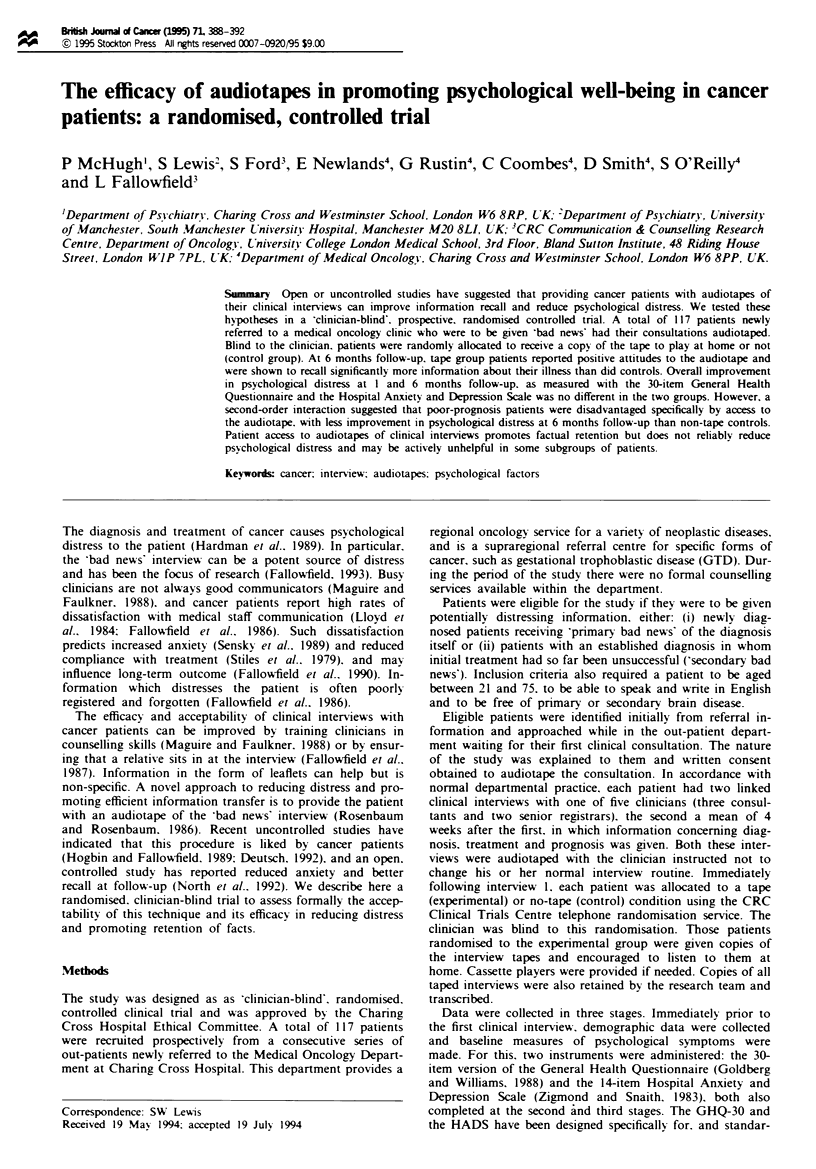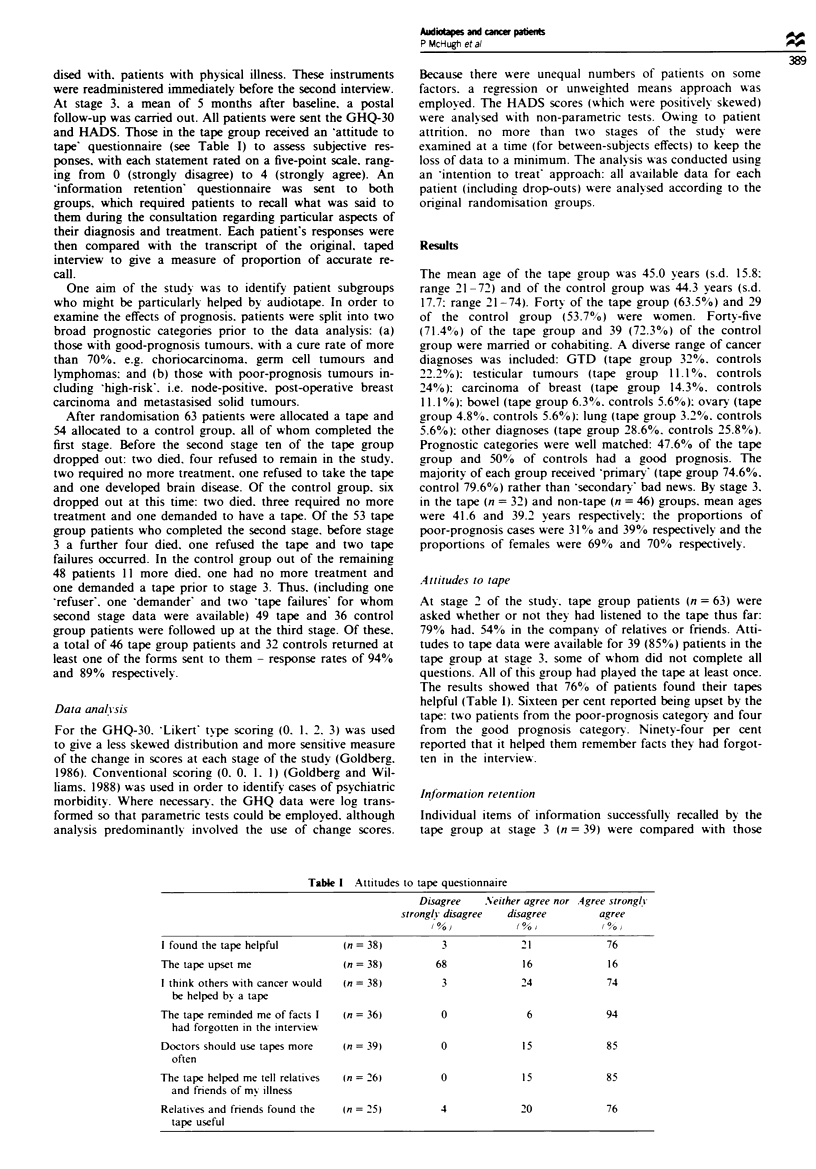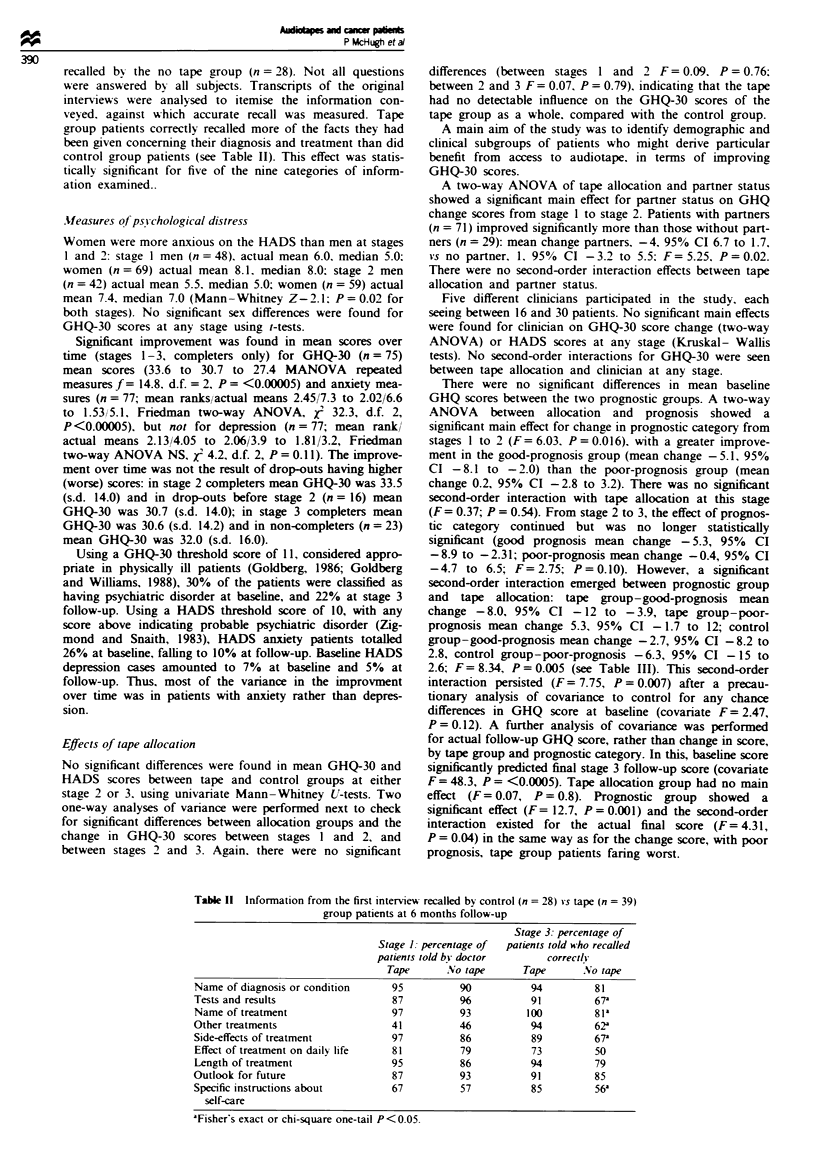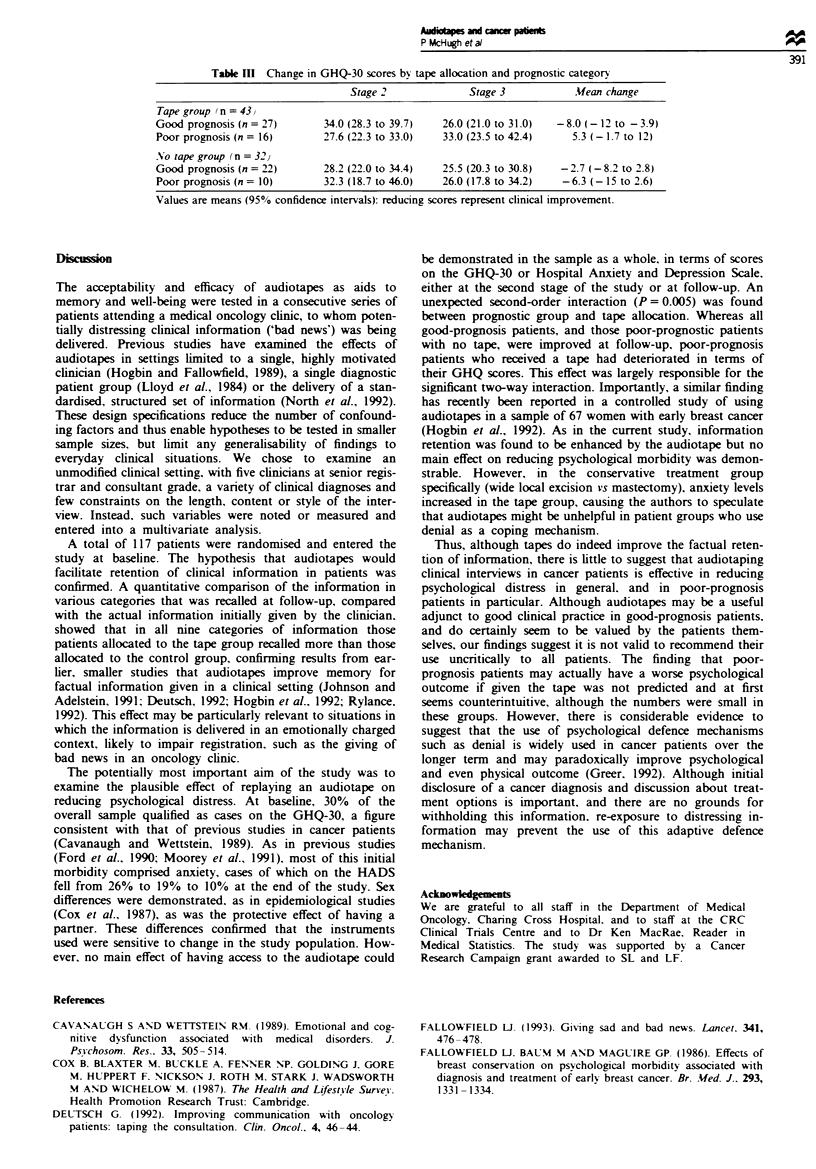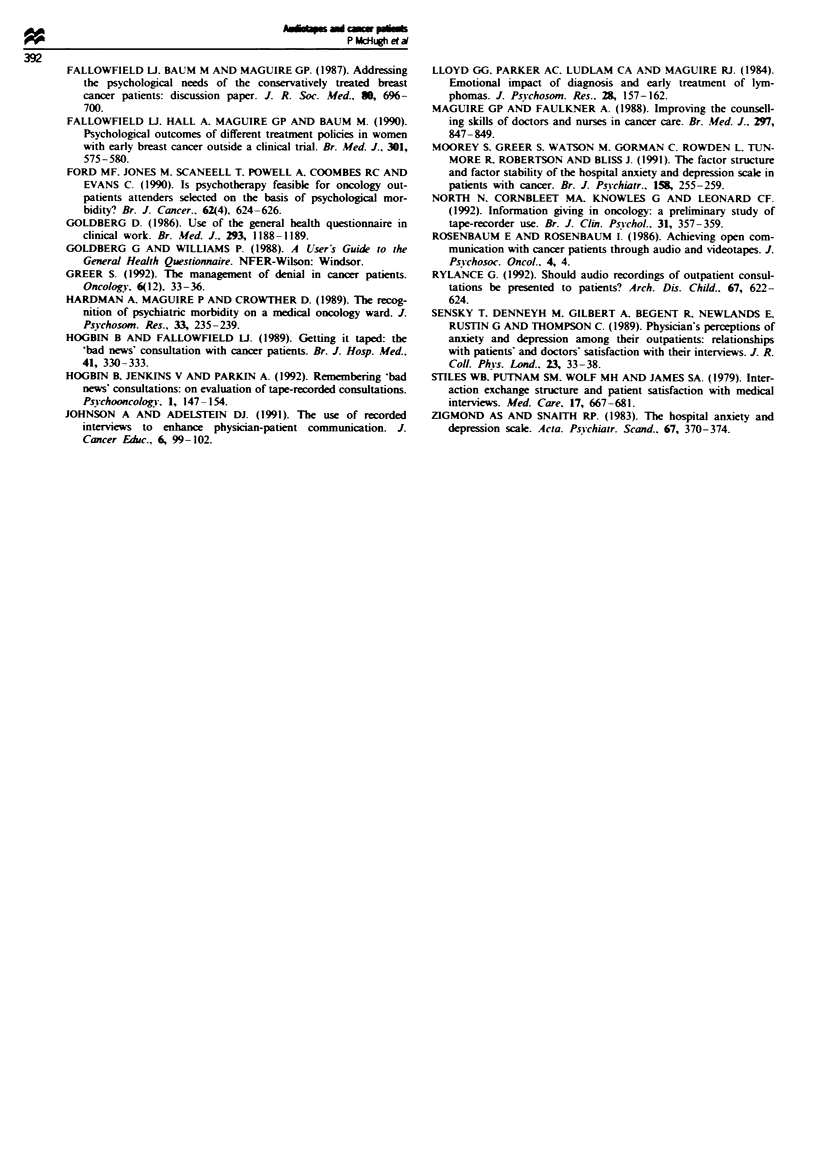# The efficacy of audiotapes in promoting psychological well-being in cancer patients: a randomised, controlled trial.

**DOI:** 10.1038/bjc.1995.79

**Published:** 1995-02

**Authors:** P. McHugh, S. Lewis, S. Ford, E. Newlands, G. Rustin, C. Coombes, D. Smith, S. O'Reilly, L. Fallowfield

**Affiliations:** Department of Psychiatry, Charing Cross and Westminster School, London, UK.

## Abstract

Open or uncontrolled studies have suggested that providing cancer patients with audiotapes of their clinical interviews can improve information recall and reduce psychological distress. We tested these hypotheses in a 'clinician-blind', prospective, randomised controlled trial. A total of 117 patients newly referred to a medical oncology clinic who were to be given 'bad news' had their consultations audiotaped. Blind to the clinician, patients were randomly allocated to receive a copy of the tape to play at home or not (control group). At 6 months follow-up, tape group patients reported positive attitudes to the audiotape and were shown to recall significantly more information about their illness than did controls. Overall improvement in psychological distress at 1 and 6 months follow-up, as measured with the 30-item General Health Questionnaire and the Hospital Anxiety and Depression Scale was no different in the two groups. However, a second-order interaction suggested that poor-prognosis patients were disadvantaged specifically by access to the audiotape, with less improvement in psychological distress at 6 months follow-up than non-tape controls. Patient access to audiotapes of clinical interviews promotes factual retention but does not reliably reduce psychological distress and may be actively unhelpful in some subgroups of patients.


British Journal of Cancer (1995) 71, 388-392

© 1995 Stockton Press All rights reserved 0007-0920/95 $9.00

### The efficacy of audiotapes in promoting psychological well-being in cancer patients: a randomised, controlled trial

P McHugh[1], S Lewis[2], S Ford[3], E Newlands[4], G Rustin[4], C Coombes[4], D Smith[4], S O'Reilly[4] and L Fallowfield[3]

[1]*Department of Psychiatry, Charing Cross and Westminster School, London W6 8RP, UK;* [2]*Department of Psychiatry, University of Manchester, South Manchester University Hospital, Manchester M20 8LI, UK;* [3]*CRC Communication & Counselling Research Centre, Department of Oncology, University College London Medical School, 3rd Floor, Bland Sutton Institute, 48 Riding House Street, London W1P 7PL, UK;* [4]*Department of Medical Oncology, Charing Cross and Westminster School, London W6 8PP, UK.*

**Summary** Open or uncontrolled studies have suggested that providing cancer patients with audiotapes of their clinical interviews can improve information recall and reduce psychological distress. We tested these hypotheses in a 'clinician-blind', prospective, randomised controlled trial. A total of 117 patients newly referred to a medical oncology clinic who were to be given 'bad news' had their consultations audiotaped. Blind to the clinician, patients were randomly allocated to receive a copy of the tape to play at home or not (control group). At 6 months follow-up, tape group patients reported positive attitudes to the audiotape and were shown to recall significantly more information about their illness than did controls. Overall improvement in psychological distress at 1 and 6 months follow-up, as measured with the 30-item General Health Questionnaire and the Hospital Anxiety and Depression Scale was no different in the two groups. However, a second-order interaction suggested that poor-prognosis patients were disadvantaged specifically by access to the audiotape, with less improvement in psychological distress at 6 months follow-up than non-tape controls. Patient access to audiotapes of clinical interviews promotes factual retention but does not reliably reduce psychological distress and may be actively unhelpful in some subgroups of patients.

**Keywords:** cancer; interview; audiotapes; psychological factors

The diagnosis and treatment of cancer causes psychological distress to the patient (Hardman *et al.*, 1989). In particular, the 'bad news' interview can be a potent source of distress and has been the focus of research (Fallowfield, 1993). Busy clinicians are not always good communicators (Maguire and Faulkner, 1988), and cancer patients report high rates of dissatisfaction with medical staff communication (Lloyd *et al.*, 1984; Fallowfield *et al.*, 1986). Such dissatisfaction predicts increased anxiety (Sensky *et al.*, 1989) and reduced compliance with treatment (Stiles *et al.*, 1979), and may influence long-term outcome (Fallowfield *et al.*, 1990). Information which distresses the patient is often poorly registered and forgotten (Fallowfield *et al.*, 1986).

The efficacy and acceptability of clinical interviews with cancer patients can be improved by training clinicians in counselling skills (Maguire and Faulkner, 1988) or by ensuring that a relative sits in at the interview (Fallowfield *et al.*, 1987). Information in the form of leaflets can help but is non-specific. A novel approach to reducing distress and promoting efficient information transfer is to provide the patient with an audiotape of the 'bad news' interview (Rosenbaum and Rosenbaum, 1986). Recent uncontrolled studies have indicated that this procedure is liked by cancer patients (Hogbin and Fallowfield, 1989; Deutsch, 1992), and an open, controlled study has reported reduced anxiety and better recall at follow-up (North *et al.*, 1992). We describe here a randomised, clinician-blind trial to assess formally the acceptability of this technique and its efficacy in reducing distress and promoting retention of facts.

### Methods

The study was designed as as 'clinician-blind', randomised, controlled clinical trial and was approved by the Charing Cross Hospital Ethical Committee. A total of 117 patients were recruited prospectively from a consecutive series of out-patients newly referred to the Medical Oncology Department at Charing Cross Hospital. This department provides a regional oncology service for a variety of neoplastic diseases, and is a supraregional referral centre for specific forms of cancer, such as gestational trophoblastic disease (GTD). During the period of the study there were no formal counselling services available within the department.

Patients were eligible for the study if they were to be given potentially distressing information, either: (i) newly diagnosed patients receiving 'primary bad news' of the diagnosis itself or (ii) patients with an established diagnosis in whom initial treatment had so far been unsuccessful ('secondary bad news'). Inclusion criteria also required a patient to be aged between 21 and 75, to be able to speak and write in English and to be free of primary or secondary brain disease.

Eligible patients were identified initially from referral information and approached while in the out-patient department waiting for their first clinical consultation. The nature of the study was explained to them and written consent obtained to audiotape the consultation. In accordance with normal departmental practice, each patient had two linked clinical interviews with one of five clinicians (three consultants and two senior registrars), the second a mean of 4 weeks after the first, in which information concerning diagnosis, treatment and prognosis was given. Both these interviews were audiotaped with the clinician instructed not to change his or her normal interview routine. Immediately following interview 1, each patient was allocated to a tape (experimental) or no-tape (control) condition using the CRC Clinical Trials Centre telephone randomisation service. The clinician was blind to this randomisation. Those patients randomised to the experimental group were given copies of the interview tapes and encouraged to listen to them at home. Cassette players were provided if needed. Copies of all taped interviews were also retained by the research team and transcribed.

Data were collected in three stages. Immediately prior to the first clinical interview, demographic data were collected and baseline measures of psychological symptoms were made. For this, two instruments were administered: the 30-item version of the General Health Questionnaire (Goldberg and Williams, 1988) and the 14-item Hospital Anxiety and Depression Scale (Zigmond and Snaith, 1983), both also completed at the second and third stages. The GHQ-30 and the HADS have been designed specifically for, and standar-

Correspondence: SW Lewis

Received 19 May 1994; accepted 19 July 1994

dised with, patients with physical illness. These instruments were readministered immediately before the second interview. At stage 3, a mean of 5 months after baseline, a postal follow-up was carried out. All patients were sent the GHQ-30 and HADS. Those in the tape group received an 'attitude to tape' questionnaire (see Table I) to assess subjective responses, with each statement rated on a five-point scale, ranging from 0 (strongly disagree) to 4 (strongly agree). An 'information retention' questionnaire was sent to both groups, which required patients to recall what was said to them during the consultation regarding particular aspects of their diagnosis and treatment. Each patient's responses were then compared with the transcript of the original, taped interview to give a measure of proportion of accurate recall.

One aim of the study was to identify patient subgroups who might be particularly helped by audiotape. In order to examine the effects of prognosis, patients were split into two broad prognostic categories prior to the data analysis: (a) those with good-prognosis tumours, with a cure rate of more than 70%, e.g. choriocarcinoma, germ cell tumours and lymphomas; and (b) those with poor-prognosis tumours including 'high-risk', i.e. node-positive, post-operative breast carcinoma and metastasised solid tumours.

After randomisation 63 patients were allocated a tape and 54 allocated to a control group, all of whom completed the first stage. Before the second stage ten of the tape group dropped out: two died, four refused to remain in the study, two required no more treatment, one refused to take the tape and one developed brain disease. Of the control group, six dropped out at this time: two died, three required no more treatment and one demanded to have a tape. Of the 53 tape group patients who completed the second stage, before stage 3 a further four died, one refused the tape and two tape failures occurred. In the control group out of the remaining 48 patients 11 more died, one had no more treatment and one demanded a tape prior to stage 3. Thus, (including one 'refuser', one 'demander' and two 'tape failures' for whom second stage data were available) 49 tape and 36 control group patients were followed up at the third stage. Of these, a total of 46 tape group patients and 32 controls returned at least one of the forms sent to them – response rates of 94% and 89% respectively.

### *Data analysis*

For the GHQ-30, 'Likert' type scoring (0, 1, 2, 3) was used to give a less skewed distribution and more sensitive measure of the change in scores at each stage of the study (Goldberg, 1986). Conventional scoring (0, 0, 1, 1) (Goldberg and Williams, 1988) was used in order to identify cases of psychiatric morbidity. Where necessary, the GHQ data were log transformed so that parametric tests could be employed, although analysis predominantly involved the use of change scores. Because there were unequal numbers of patients on some factors, a regression or unweighted means approach was employed. The HADS scores (which were positively skewed) were analysed with non-parametric tests. Owing to patient attrition, no more than two stages of the study were examined at a time (for between-subjects effects) to keep the loss of data to a minimum. The analysis was conducted using an 'intention to treat' approach: all available data for each patient (including drop-outs) were analysed according to the original randomisation groups.

### Results

The mean age of the tape group was 45.0 years (s.d. 15.8; range 21–72) and of the control group was 44.3 years (s.d. 17.7; range 21–74). Forty of the tape group (63.5%) and 29 of the control group (53.7%) were women. Forty-five (71.4%) of the tape group and 39 (72.3%) of the control group were married or cohabiting. A diverse range of cancer diagnoses was included: GTD (tape group 32%, controls 22.2%); testicular tumours (tape group 11.1%, controls 24%); carcinoma of breast (tape group 14.3%, controls 11.1%); bowel (tape group 6.3%, controls 5.6%); ovary (tape group 4.8%, controls 5.6%); lung (tape group 3.2%, controls 5.6%); other diagnoses (tape group 28.6%, controls 25.8%). Prognostic categories were well matched: 47.6% of the tape group and 50% of controls had a good prognosis. The majority of each group received 'primary' (tape group 74.6%, control 79.6%) rather than 'secondary' bad news. By stage 3, in the tape ($n = 32$) and non-tape ($n = 46$) groups, mean ages were 41.6 and 39.2 years respectively; the proportions of poor-prognosis cases were 31% and 39% respectively and the proportions of females were 69% and 70% respectively.

### *Attitudes to tape*

At stage 2 of the study, tape group patients ($n = 63$) were asked whether or not they had listened to the tape thus far: 79% had, 54% in the company of relatives or friends. Attitudes to tape data were available for 39 (85%) patients in the tape group at stage 3, some of whom did not complete all questions. All of this group had played the tape at least once. The results showed that 76% of patients found their tapes helpful (Table I). Sixteen per cent reported being upset by the tape: two patients from the poor-prognosis category and four from the good prognosis category. Ninety-four per cent reported that it helped them remember facts they had forgotten in the interview.

### *Information retention*

Individual items of information successfully recalled by the tape group at stage 3 ($n = 39$) were compared with those

**Table I** Attitudes to tape questionnaire

| | | *Disagree strongly disagree (%)* | *Neither agree nor disagree (%)* | *Agree strongly agree (%)* |
|---|---|---|---|---|
| I found the tape helpful | ($n = 38$) | 3 | 21 | 76 |
| The tape upset me | ($n = 38$) | 68 | 16 | 16 |
| I think others with cancer would be helped by a tape | ($n = 38$) | 3 | 24 | 74 |
| The tape reminded me of facts I had forgotten in the interview | ($n = 36$) | 0 | 6 | 94 |
| Doctors should use tapes more often | ($n = 39$) | 0 | 15 | 85 |
| The tape helped me tell relatives and friends of my illness | ($n = 26$) | 0 | 15 | 85 |
| Relatives and friends found the tape useful | ($n = 25$) | 4 | 20 | 76 |

recalled by the no tape group ($n = 28$). Not all questions were answered by all subjects. Transcripts of the original interviews were analysed to itemise the information conveyed, against which accurate recall was measured. Tape group patients correctly recalled more of the facts they had been given concerning their diagnosis and treatment than did control group patients (see Table II). This effect was statistically significant for five of the nine categories of information examined..

### *Measures of psychological distress*

Women were more anxious on the HADS than men at stages 1 and 2: stage 1 men ($n = 48$), actual mean 6.0, median 5.0; women ($n = 69$) actual mean 8.1, median 8.0; stage 2 men ($n = 42$) actual mean 5.5, median 5.0; women ($n = 59$) actual mean 7.4, median 7.0 (Mann–Whitney $Z - 2.1$; $P = 0.02$ for both stages). No significant sex differences were found for GHQ-30 scores at any stage using $t$-tests.

Significant improvement was found in mean scores over time (stages 1–3, completers only) for GHQ-30 ($n = 75$) mean scores (33.6 to 30.7 to 27.4 MANOVA repeated measures $f = 14.8$, d.f. = 2, $P = <0.00005$) and anxiety measures ($n = 77$; mean ranks/actual means 2.45/7.3 to 2.02/6.6 to 1.53/5.1, Friedman two-way ANOVA, $\chi^2$ 32.3, d.f. 2, $P<0.00005$), but *not* for depression ($n = 77$; mean rank/actual means 2.13/4.05 to 2.06/3.9 to 1.81/3.2, Friedman two-way ANOVA NS, $\chi^2$ 4.2, d.f. 2, $P = 0.11$). The improvement over time was not the result of drop-outs having higher (worse) scores: in stage 2 completers mean GHQ-30 was 33.5 (s.d. 14.0) and in drop-outs before stage 2 ($n = 16$) mean GHQ-30 was 30.7 (s.d. 14.0); in stage 3 completers mean GHQ-30 was 30.6 (s.d. 14.2) and in non-completers ($n = 23$) mean GHQ-30 was 32.0 (s.d. 16.0).

Using a GHQ-30 threshold score of 11, considered appropriate in physically ill patients (Goldberg, 1986; Goldberg and Williams, 1988), 30% of the patients were classified as having psychiatric disorder at baseline, and 22% at stage 3 follow-up. Using a HADS threshold score of 10, with any score above indicating probable psychiatric disorder (Zigmond and Snaith, 1983), HADS anxiety patients totalled 26% at baseline, falling to 10% at follow-up. Baseline HADS depression cases amounted to 7% at baseline and 5% at follow-up. Thus, most of the variance in the improvment over time was in patients with anxiety rather than depression.

### *Effects of tape allocation*

No significant differences were found in mean GHQ-30 and HADS scores between tape and control groups at either stage 2 or 3, using univariate Mann–Whitney $U$-tests. Two one-way analyses of variance were performed next to check for significant differences between allocation groups and the change in GHQ-30 scores between stages 1 and 2, and between stages 2 and 3. Again, there were no significant differences (between stages 1 and 2 $F = 0.09$, $P = 0.76$; between 2 and 3 $F = 0.07$, $P = 0.79$), indicating that the tape had no detectable influence on the GHQ-30 scores of the tape group as a whole, compared with the control group.

A main aim of the study was to identify demographic and clinical subgroups of patients who might derive particular benefit from access to audiotape, in terms of improving GHQ-30 scores.

A two-way ANOVA of tape allocation and partner status showed a significant main effect for partner status on GHQ change scores from stage 1 to stage 2. Patients with partners ($n = 71$) improved significantly more than those without partners ($n = 29$): mean change partners, −4, 95% CI 6.7 to 1.7, *vs* no partner, 1, 95% CI −3.2 to 5.5; $F = 5.25$, $P = 0.02$. There were no second-order interaction effects between tape allocation and partner status.

Five different clinicians participated in the study, each seeing between 16 and 30 patients. No significant main effects were found for clinician on GHQ-30 score change (two-way ANOVA) or HADS scores at any stage (Kruskal– Wallis tests). No second-order interactions for GHQ-30 were seen between tape allocation and clinician at any stage.

There were no significant differences in mean baseline GHQ scores between the two prognostic groups. A two-way ANOVA between allocation and prognosis showed a significant main effect for change in prognostic category from stages 1 to 2 ($F = 6.03$, $P = 0.016$), with a greater improvement in the good-prognosis group (mean change −5.1, 95% CI −8.1 to −2.0) than the poor-prognosis group (mean change 0.2, 95% CI −2.8 to 3.2). There was no significant second-order interaction with tape allocation at this stage ($F = 0.37$; $P = 0.54$). From stage 2 to 3, the effect of prognostic category continued but was no longer statistically significant (good prognosis mean change −5.3, 95% CI −8.9 to −2.31; poor-prognosis mean change −0.4, 95% CI −4.7 to 6.5; $F = 2.75$; $P = 0.10$). However, a significant second-order interaction emerged between prognostic group and tape allocation: tape group–good-prognosis mean change −8.0, 95% CI −12 to −3.9, tape group–poor-prognosis mean change 5.3, 95% CI −1.7 to 12; control group–good-prognosis mean change −2.7, 95% CI −8.2 to 2.8, control group–poor-prognosis −6.3, 95% CI −15 to 2.6; $F = 8.34$, $P = 0.005$ (see Table III). This second-order interaction persisted ($F = 7.75$, $P = 0.007$) after a precautionary analysis of covariance to control for any chance differences in GHQ score at baseline (covariate $F = 2.47$, $P = 0.12$). A further analysis of covariance was performed for actual follow-up GHQ score, rather than change in score, by tape group and prognostic category. In this, baseline score significantly predicted final stage 3 follow-up score (covariate $F = 48.3$, $P = <0.0005$). Tape allocation group had no main effect ($F = 0.07$, $P = 0.8$). Prognostic group showed a significant effect ($F = 12.7$, $P = 0.001$) and the second-order interaction existed for the actual final score ($F = 4.31$, $P = 0.04$) in the same way as for the change score, with poor prognosis, tape group patients faring worst.

**Table II** Information from the first interview recalled by control ($n = 28$) *vs* tape ($n = 39$) group patients at 6 months follow-up

| | *Stage 1: percentage of patients told by doctor* | | *Stage 3: percentage of patients told who recalled correctly* | |
|---|---|---|---|---|
| | *Tape* | *No tape* | *Tape* | *No tape* |
| Name of diagnosis or condition | 95 | 90 | 94 | 81 |
| Tests and results | 87 | 96 | 91 | 67[a] |
| Name of treatment | 97 | 93 | 100 | 81[a] |
| Other treatments | 41 | 46 | 94 | 62[a] |
| Side-effects of treatment | 97 | 86 | 89 | 67[a] |
| Effect of treatment on daily life | 81 | 79 | 73 | 50 |
| Length of treatment | 95 | 86 | 94 | 79 |
| Outlook for future | 87 | 93 | 91 | 85 |
| Specific instructions about self-care | 67 | 57 | 85 | 56[a] |

[a]Fisher's exact or chi-square one-tail $P<0.05$.

Table III Change in GHQ-30 scores by tape allocation and prognostic category

| | Stage 2 | Stage 3 | Mean change |
|---|---|---|---|
| *Tape group* (n = *43*) | | | |
| Good prognosis ($n$ = 27) | 34.0 (28.3 to 39.7) | 26.0 (21.0 to 31.0) | − 8.0 ( − 12 to − 3.9) |
| Poor prognosis ($n$ = 16) | 27.6 (22.3 to 33.0) | 33.0 (23.5 to 42.4) | 5.3 ( − 1.7 to 12) |
| *No tape group* (n = *32*) | | | |
| Good prognosis ($n$ = 22) | 28.2 (22.0 to 34.4) | 25.5 (20.3 to 30.8) | − 2.7 ( − 8.2 to 2.8) |
| Poor prognosis ($n$ = 10) | 32.3 (18.7 to 46.0) | 26.0 (17.8 to 34.2) | − 6.3 ( − 15 to 2.6) |

Values are means (95% confidence intervals): reducing scores represent clinical improvement.

### Discussion

The acceptability and efficacy of audiotapes as aids to memory and well-being were tested in a consecutive series of patients attending a medical oncology clinic, to whom potentially distressing clinical information ('bad news') was being delivered. Previous studies have examined the effects of audiotapes in settings limited to a single, highly motivated clinician (Hogbin and Fallowfield, 1989), a single diagnostic patient group (Lloyd *et al.*, 1984) or the delivery of a standardised, structured set of information (North *et al.*, 1992). These design specifications reduce the number of confounding factors and thus enable hypotheses to be tested in smaller sample sizes, but limit any generalisability of findings to everyday clinical situations. We chose to examine an unmodified clinical setting, with five clinicians at senior registrar and consultant grade, a variety of clinical diagnoses and few constraints on the length, content or style of the interview. Instead, such variables were noted or measured and entered into a multivariate analysis.

A total of 117 patients were randomised and entered the study at baseline. The hypothesis that audiotapes would facilitate retention of clinical information in patients was confirmed. A quantitative comparison of the information in various categories that was recalled at follow-up, compared with the actual information initially given by the clinician, showed that in all nine categories of information those patients allocated to the tape group recalled more than those allocated to the control group, confirming results from earlier, smaller studies that audiotapes improve memory for factual information given in a clinical setting (Johnson and Adelstein, 1991; Deutsch, 1992; Hogbin *et al.*, 1992; Rylance, 1992). This effect may be particularly relevant to situations in which the information is delivered in an emotionally charged context, likely to impair registration, such as the giving of bad news in an oncology clinic.

The potentially most important aim of the study was to examine the plausible effect of replaying an audiotape on reducing psychological distress. At baseline, 30% of the overall sample qualified as cases on the GHQ-30, a figure consistent with that of previous studies in cancer patients (Cavanaugh and Wettstein, 1989). As in previous studies (Ford *et al.*, 1990; Moorey *et al.*, 1991), most of this initial morbidity comprised anxiety, cases of which on the HADS fell from 26% to 19% to 10% at the end of the study. Sex differences were demonstrated, as in epidemiological studies (Cox *et al.*, 1987), as was the protective effect of having a partner. These differences confirmed that the instruments used were sensitive to change in the study population. However, no main effect of having access to the audiotape could be demonstrated in the sample as a whole, in terms of scores on the GHQ-30 or Hospital Anxiety and Depression Scale, either at the second stage of the study or at follow-up. An unexpected second-order interaction ($P = 0.005$) was found between prognostic group and tape allocation. Whereas all good-prognosis patients, and those poor-prognostic patients with no tape, were improved at follow-up, poor-prognosis patients who received a tape had deteriorated in terms of their GHQ scores. This effect was largely responsible for the significant two-way interaction. Importantly, a similar finding has recently been reported in a controlled study of using audiotapes in a sample of 67 women with early breast cancer (Hogbin *et al.*, 1992). As in the current study, information retention was found to be enhanced by the audiotape but no main effect on reducing psychological morbidity was demonstrable. However, in the conservative treatment group specifically (wide local excision *vs* mastectomy), anxiety levels increased in the tape group, causing the authors to speculate that audiotapes might be unhelpful in patient groups who use denial as a coping mechanism.

Thus, although tapes do indeed improve the factual retention of information, there is little to suggest that audiotaping clinical interviews in cancer patients is effective in reducing psychological distress in general, and in poor-prognosis patients in particular. Although audiotapes may be a useful adjunct to good clinical practice in good-prognosis patients, and do certainly seem to be valued by the patients themselves, our findings suggest it is not valid to recommend their use uncritically to all patients. The finding that poor-prognosis patients may actually have a worse psychological outcome if given the tape was not predicted and at first seems counterintuitive, although the numbers were small in these groups. However, there is considerable evidence to suggest that the use of psychological defence mechanisms such as denial is widely used in cancer patients over the longer term and may paradoxically improve psychological and even physical outcome (Greer, 1992). Although initial disclosure of a cancer diagnosis and discussion about treatment options is important, and there are no grounds for withholding this information, re-exposure to distressing information may prevent the use of this adaptive defence mechanism.

### Acknowledgements
We are grateful to all staff in the Department of Medical Oncology, Charing Cross Hospital, and to staff at the CRC Clinical Trials Centre and to Dr Ken MacRae, Reader in Medical Statistics. The study was supported by a Cancer Research Campaign grant awarded to SL and LF.

### References

CAVANAUGH S AND WETTSTEIN RM. (1989). Emotional and cognitive dysfunction associated with medical disorders. *J. Psychosom. Res.*, **33**, 505–514.

COX B, BLAXTER M, BUCKLE A, FENNER NP, GOLDING J, GORE M, HUPPERT F, NICKSON J, ROTH M, STARK J, WADSWORTH M AND WICHELOW M. (1987). *The Health and Lifestyle Survey*. Health Promotion Research Trust: Cambridge.

DEUTSCH G. (1992). Improving communication with oncology patients: taping the consultation. *Clin. Oncol.*, **4**, 46–44.

FALLOWFIELD LJ. (1993). Giving sad and bad news. *Lancet*, **341**, 476–478.

FALLOWFIELD LJ, BAUM M AND MAGUIRE GP. (1986). Effects of breast conservation on psychological morbidity associated with diagnosis and treatment of early breast cancer. *Br. Med. J.*, **293**, 1331–1334.

FALLOWFIELD LJ, BAUM M AND MAGUIRE GP. (1987). Addressing the psychological needs of the conservatively treated breast cancer patients: discussion paper. *J. R. Soc. Med.*, **80**, 696–700.

FALLOWFIELD LJ, HALL A, MAGUIRE GP AND BAUM M. (1990). Psychological outcomes of different treatment policies in women with early breast cancer outside a clinical trial. *Br. Med. J.*, **301**, 575–580.

FORD MF, JONES M, SCANEELL T, POWELL A, COOMBES RC AND EVANS C. (1990). Is psychotherapy feasible for oncology outpatients attenders selected on the basis of psychological morbidity? *Br. J. Cancer*, **62**(4), 624–626.

GOLDBERG D. (1986). Use of the general health questionnaire in clinical work. *Br. Med. J.*, **293**, 1188–1189.

GOLDBERG G AND WILLIAMS P. (1988). *A User's Guide to the General Health Questionnaire*. NFER-Wilson: Windsor.

GREER S. (1992). The management of denial in cancer patients. *Oncology*, **6**(12), 33–36.

HARDMAN A, MAGUIRE P AND CROWTHER D. (1989). The recognition of psychiatric morbidity on a medical oncology ward. *J. Psychosom. Res.*, **33**, 235–239.

HOGBIN B AND FALLOWFIELD LJ. (1989). Getting it taped: the 'bad news' consultation with cancer patients. *Br. J. Hosp. Med.*, **41**, 330–333.

HOGBIN B, JENKINS V AND PARKIN A. (1992). Remembering 'bad news' consultations: on evaluation of tape-recorded consultations. *Psychooncology*, **1**, 147–154.

JOHNSON A AND ADELSTEIN DJ. (1991). The use of recorded interviews to enhance physician-patient communication. *J. Cancer Educ.*, **6**, 99–102.

LLOYD GG, PARKER AC, LUDLAM CA AND MAGUIRE RJ. (1984). Emotional impact of diagnosis and early treatment of lymphomas. *J. Psychosom. Res.*, **28**, 157–162.

MAGUIRE GP AND FAULKNER A. (1988). Improving the counselling skills of doctors and nurses in cancer care. *Br. Med. J.*, **297**, 847–849.

MOOREY S, GREER S, WATSON M, GORMAN C, ROWDEN L, TUNMORE R, ROBERTSON AND BLISS J. (1991). The factor structure and factor stability of the hospital anxiety and depression scale in patients with cancer. *Br. J. Psychiatr.*, **158**, 255–259.

NORTH N, CORNBLEET MA, KNOWLES G AND LEONARD CF. (1992). Information giving in oncology: a preliminary study of tape-recorder use. *Br. J. Clin. Psychol.*, **31**, 357–359.

ROSENBAUM E AND ROSENBAUM I. (1986). Achieving open communication with cancer patients through audio and videotapes. *J. Psychosoc. Oncol.*, **4**, 4.

RYLANCE G. (1992). Should audio recordings of outpatient consultations be presented to patients? *Arch. Dis. Child.*, **67**, 622–624.

SENSKY T, DENNEYH M, GILBERT A, BEGENT R, NEWLANDS E, RUSTIN G AND THOMPSON C. (1989). Physician's perceptions of anxiety and depression among their outpatients: relationships with patients' and doctors' satisfaction with their interviews. *J. R. Coll. Phys. Lond.*, **23**, 33–38.

STILES WB, PUTNAM SM, WOLF MH AND JAMES SA. (1979). Interaction exchange structure and patient satisfaction with medical interviews. *Med. Care*, **17**, 667–681.

ZIGMOND AS AND SNAITH RP. (1983). The hospital anxiety and depression scale. *Acta. Psychiatr. Scand.*, **67**, 370–374.